# High Endogenously Synthesized N-3 Polyunsaturated Fatty Acids in Fat-1 Mice Attenuate High-Fat Diet-Induced Insulin Resistance by Inhibiting NLRP3 Inflammasome Activation via Akt/GSK-3β/TXNIP Pathway

**DOI:** 10.3390/molecules27196384

**Published:** 2022-09-27

**Authors:** Pan Zhu, Jin-Jie Zhang, Yi Cen, Yong Yang, Feng Wang, Kun-Peng Gu, Hai-Tao Yang, Yun-Zhi Wang, Zu-Quan Zou

**Affiliations:** 1 Ningbo Beilun District for Disease Control and Prevention, Ningbo 315000, China; 2 College of Food and Pharmaceutical Sciences, Ningbo University, Ningbo 315211, China; 3 Department of Clinical Laboratory, The Affiliated Hospital of Ningbo University, Ningbo 315020, China; 4 Department of Laboratory Medicine, Ningbo Medical Center Lihuili Hospital, Ningbo 315048, China; 5 Department of Pathology, Mingzhou Hospital of Zhejiang University, Ningbo 315199, China; 6 School of Health Sciences, University of Sydney, P.O. Box 170, Lidcombe, Sydney, NSW 1825, Australia; 7 School of Public Health, Ningbo University, Ningbo 315211, China

**Keywords:** obesity, insulin resistance, NLRP3 inflammasome, IL-1β, n-3 PUFAs, docosahexaenoic acid, fat-1

## Abstract

High-fat (HF) diets and low-grade chronic inflammation contribute to the development of insulin resistance and type 2 diabetes (T2D), whereas n-3 polyunsaturated fatty acids (PUFAs), due to their anti-inflammatory effects, protect against insulin resistance. Interleukin (IL)-1β is implicated in insulin resistance, yet how n-3 PUFAs modulate IL-1β secretion and attenuate HF diet-induced insulin resistance remains elusive. In this study, a HF diet activated NLRP3 inflammasome via inducing reactive oxygen species (ROS) generation and promoted IL-1β production primarily from adipose tissue preadipocytes, but not from adipocytes and induced insulin resistance in wild type (WT) mice. Interestingly, endogenous synthesized n-3 polyunsaturated fatty acids (PUFAs) reversed this process in HF diet-fed fat-1 transgenic mice although the HF diet induced higher weight gain in fat-1 mice, compared with the control diet. Mechanistically, palmitic acid (PA), the main saturated fatty acid in an HF diet inactivated AMPK and led to decreased GSK-3β phosphorylation, at least partially through reducing Akt activity, which ultimately blocked the Nrf2/Trx1 antioxidant pathway and induced TXNIP cytoplasm translocation and NLRP3 inflammasome activation, whereas docosahexaenoic acid (DHA), the most abundant n-3 PUFA in fat-1 adipose tissue, reversed this process via inducing Akt activation. Our *GSK-3β* shRNA knockdown study further revealed that GSK-3β played a pivot role between the upstream AMPK/Akt pathway and downstream Nrf2/Trx1/TXNIP pathway. Given that NLRP3 inflammasome is implicated in the development of most inflammatory diseases, our results suggest the potential of n-3 PUFAs in the prevention or adjuvant treatment of NLRP3 inflammasome-driven diseases.

## 1. Introduction

The global Obesity rate has increased in the past 50 years, with estimates that nearly a third of the world’s population is now classified as overweight or obese. Obesity substantially increases the risk of metabolic diseases including type-2 diabetes (T2D), cardiovascular disease, nonalcoholic fatty liver disease and several forms of cancer [1]. Adipose tissue is recognized as not only simply a fat-storing organelle, thermal regulator, or protective padding for important organs but also a metabolically active endocrine organ secreting a variety of hormones, peptides and metabolites [2]. Among these pro-inflammatory cytokines and adipokines secreted by adipose tissue, interleukin (IL)-1β plays a critical role in chronic low-grade inflammation in adipose tissue and is in part responsible for the development of insulin resistance [3,4]. IL-1β, one of the IL-1 family cytokines, is tightly regulated by the NLR family pyrin domain-containing 3 (NLRP3) inflammasome consisting of a pattern recognition receptor (PRR), the apoptosis-associated speck-like protein containing a CARD (ASC/PYCARD), and caspase-1 [5]. IL-1β release requires a two-phase process. The first phase is a prime step to produce pro-IL-1β via Toll-like receptor 4 (TLR4)/nuclear factor-kB. The NLRP3 inflammasome, which is activated by danger-associated molecular pattern molecules such as ATP, uric acid, reactive oxygen species (ROS) or ceramides, is involved in the second phase, an activation step designed to produce mature IL-1β [6].

Numerous prospective epidemiological studies have reported an association between dietary fats and low-grade inflammation [7,8]. The amount and type of dietary fat in the diet contribute diverse effects on regulating adipose tissue inflammatory and immune responses [9]. Palmitic acid (PA), the main saturated fatty acid in a high-fat (HF) diet but not the obese phenotype itself activates the TLR4 and is responsible for the increased release of IL-1β [10]. Recent studies in vitro and in animal models have shown that long-chain (n-3) polyunsaturated fatty acids (PUFAs) of marine origin, namely eicosapentaenoic acid (EPA) and docosahexaenoic acid (DHA), have the potential for improving adipose tissue inflammation and modulating adipose tissue function [11,12]. For instance, Weldon et al. found that pretreatment of human macrophages with EPA or DHA reduced the LPS-stimulated expression of pro-IL-1β and secretion of mature IL-1β [13]. However, the underlying mechanism by which n-3 PUFAs modulate NLRP3 inflammasome activity remains elusive. Of note, there are some discrepancies about the assumption of n-3 PUFAs possessing anti-inflammatory properties. For example, Paschoal et al. found that DHA induced the production of IL-1β in the presence of LPS [14]. Similarly, Han et al. found that DHA enhanced the levels of pro-IL-1β in the spleen of immunosuppressive mice [15]. Many potential confounding factors such as potential variations in impurities, calories, other bioactive compounds and susceptibility to oxidation in the supplemented diets might have been responsible for the above-mentioned discrepancies. Therefore, an appropriate animal model that eliminates potential dietary confounding factors is ideal for identifying the role of n-3 PUFAs in modulating NLRP3 inflammasome activity and metabolic dysfunction. The fat-1 transgenic mice carrying the *fat-1* gene, which encodes an n-3 PUFA desaturase, are capable of catalyzing the conversion of n-6 to n-3 PUFAs, leading to increased n-3 PUFAs and a balanced ratio of n-6/n-3 PUFAs in their tissues and organs compared to their WT littermates maintained on an identical diet (high in n-6 PUFAs), without the need of a dietary n-3 supplement [16]. Thus, the fat-1 transgenic mice are widely used to investigate the benefits of n-3 PUFAs and their underlying molecular mechanisms [17,18,19,20,21,22].

In the present study, the fat-1 transgenic mice were used to study the role of n-3 PUFAs in regulating the NLRP3 inflammasome activity and their underlying mechanisms. It was found that endogenously synthesized n-3 PUFA blocked HF diet-induced NLRP3 inflammasome activation and the release of IL-1β in adipose tissue, stromal vascular fraction (SVF) and adipose tissue preadipocytes, which attenuated insulin resistance in HF diet-fed fat-1 mice. Our in vitro study revealed that palmitic acid (PA) suppressed AMP-activated protein kinase (AMPK) phosphorylation, which activated glycogen synthase kinase-3β (GSK-3β) at least partly via reducing Akt phosphorylation, blocked the transcription factor nuclear factor-erythroid 2-related factor 2 (Nrf2)/thioredoxin-1 (Trx1) antioxidant system and promoted the cytoplasmic translocation of thioredoxin-interacting protein (TXNIP), leading to NLRP3 inflammasome activation and IL-1β release, whereas the process was reversed by DHA via activating Akt and inactivating GSK-3β.

## 2. Results

### 2.1. Fat-1 Trangenic Mice Exhibited Ameliorated Glucose Metabolism Disturbance Induced by HF Diet

**Figure 1:**
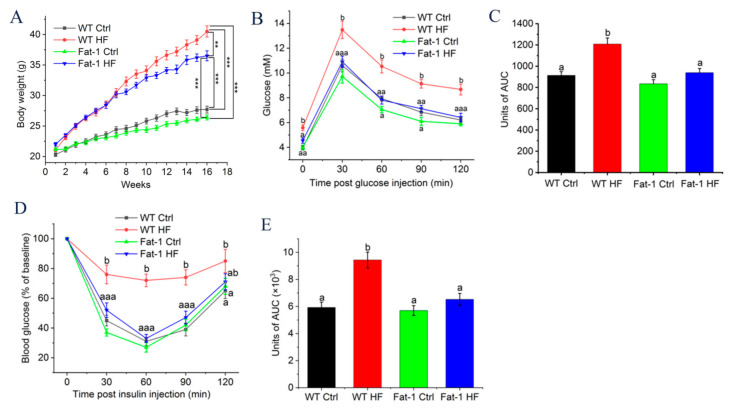
Less weight gain and improved glucose tolerance and insulin sensitivity, and lowered hyperglycemia levels were shown in HF diet-fed fat-1 mice: (**A**) Effects of endogenous n-3 PUFAs on body weight gain (*n* = 8); (**B**) OGTT was performed in fasted WT and fat-1 transgenic animals (*n* = 5); (**C**) Area under the curve (AUC) of the mean glucose levels after OGTT; (**D**) ITT was performed and normalized for base blood glucose in fasted animals (*n* = 5); (**E**) The total area under the curve (AUC). ** *p* < 0.01 and *** *p* < 0.001. Means not sharing a common superscript letter are significantly different at *p* < 0.05. In addition, because there was little difference in glucose level among group WT Ctrl, Fat-1 Ctrl and Fat-1 HF in 30 and 120 min after glucose injection in (**B**), so superscript letter of these three groups was labelled together such as aaa, which means no significant difference among group WT Ctrl, Fat-1 Ctrl and Fat-1 HF, and so on aa is similar to aaa. WT Ctrl, WT mice fed a control diet; WT HF, WT mice fed a high-fat diet; Fat-1 Ctrl, fat-1 mice fed a control diet; Fat-1HF, fat-1 mice fed a high-fat diet.

As shown in Figure 1A, compared to the Ctrl diet, the HF diet induced a statistically significant weight gain in fat-1 and WT mice although weight gain was relatively lower in fat-1 mice. Obesity is known to be associated with impaired glucose tolerance. Our results showed that HF diet-fed fat-1 (Fat-1 HF) mice had significantly lower fasting glucose, and were also protected against impaired glucose tolerance observed in HF diet-fed WT (WT HF) mice (Figure 1B,C). In accordance with this remarkable resistance to impaired glucose tolerance, the insulin tolerance test (ITT) showed that compared with WT HF mice, Fat-1 HF mice elicited a significant improvement in insulin sensitivity and remained comparable to Ctrl diet-fed mice (Figure 1D,E).

### 2.2. Fat-1 Mice Were Protected against HF Diet-Mediated IL-1β Secretion in Adipose Tissue

**Figure 2:**
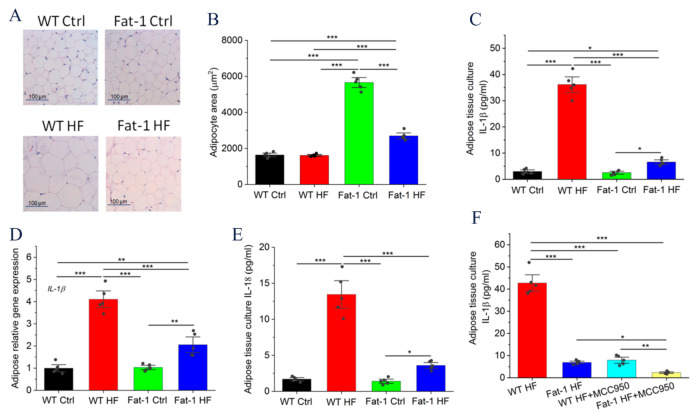
Endogenous n-3 PUFA suppressed HF diet-induced IL-1β and IL-18 secretion in adipose tissue: (**A**) Adipocyte size was monitored in adipose tissue samples by hematoxylin-eosin staining; (**B**) Average adipocyte size of white adipose tissue; (**C**) Adipose tissue from WT Ctrl, WT HF, Fat-1 Ctrl and Fat-1 HF mice was cultured in opti-MEM medium (100 mg/mL) for 24 h and IL-1β released into culture media was measured by ELISA (*n* = 5); (**D**) *IL-1β* mRNA levels in adipose tissue from WT Ctrl, WT HF, Fat-1 Ctrl and Fat-1 HF mice were detected by RT-PCR; (**E**) Adipose tissue from WT Ctrl, WT HF, Fat-1 Ctrl and Fat-1 HF mice was cultured in opti-MEM medium for 24 h and IL-18 released into culture media was measured by ELISA (*n* = 5); (**F**) Adipose tissue from WT HF and Fat-1 HF mice was cultured in opti-MEM medium for 24 h with or without MCC950 (NLRP3 inflammasome inhibitor) and IL-1β released into culture media was measured by ELISA (*n* = 5). Results are presented as mean ± SEM, * *p* < 0.05, ** *p* < 0.01 and *** *p* < 0.001.

Adipose tissue H&E staining showed that Fat-1 HF mice displayed adipose hyperplasia compared with WT HF mice, the adipocyte size from the WT HF group was much larger than that from the Fat-1HF group and control-diet group (Figure 2A,B). It is well known that obesity is closely associated with inflammation, especially in promoting the secretion of pro-inflammatory cytokines [14]. Our results demonstrated that WT HF mice displayed increased IL-1β secretion to a greater extent in adipose tissue than Fat-1 HF mice. The culture media concentration of IL-1β was (36.12 ± 1.98) and (6.56 + 0.57) pg/mL in WT HF and Fat-1 HF adipose tissue, respectively (Figure 2C). As shown in Figure 2D, adipose tissue *IL-1β* mRNA expression was also significantly upregulated in WT HF mice compared with Fat-1 HF mice. As expected, the HF diet significantly induced IL-18 secretion from adipose tissue in WT mice compared with fat-1 mice (Figure 2E). To test whether IL-1β secretion was mediated by NLRP3 inflammasome, adipose tissue was treated with NLRP3 inflammasome inhibitor MCC950. Indeed, MCC950 significantly decreased IL-1β levels in WT HF mice to an extent comparable to those in Fat-1 HF mice, suggesting that NLRP3 inflammasome is involved in controlling IL-1β secretion (Figure 2F).

### 2.3. Fat-1 HF Mice Exhibited Attenuated NLRP3 Inflammasome Activity and IL-1β Secretion in Adipose Tissue Stromal Vascular Fraction (SVF) and Preadipocytes

**Figure 3:**
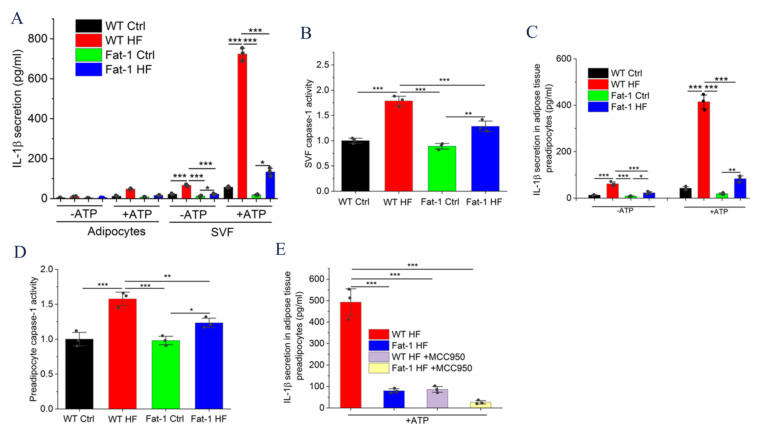
Endogenous n-3 PUFAs blocked caspase-1 activity and IL-1β secretion from SVF or preadipocytes of adipose tissue isolated from Fat-1 HF mice: (**A**) Adipocytes and SVF were isolated from epididymal fat pads by collagenase digestion. IL-1β release from the SVF (1 × 10^6^ cells/mL) and adipocytes (200 μL packed volume/mL) cultured in opti-MEM medium for 24 h with or without ATP (5 mmol/L) was measured by ELISA; (**B**) Caspase-1 activity was detected in the SVF treated with ATP (5 mmol/L) for 24 h; (**C**) Preadipocytes isolated from adipose tissue were cultured in opti-MEM medium for 24 h with or without ATP (5 mmol/L) and IL-1β release was detected in culture media by ELISA; (**D**) Caspase-1 activity was detected in the preadipocytes treated with ATP (5 mmol/L) for 24 h; (**E**) Preadipocytes isolated from adipose tissue of WT HF and Fat-1 HF were cultured in opti-MEM medium for 24 h with or without MCC950 (NLRP3 inflammasome inhibitor) and IL-1β released into culture media was measured by ELISA. Results are presented as mean ± SEM, * *p* < 0.05, ** *p* < 0.01 and *** *p* < 0.001.

We next studied which cells in adipose tissue control IL-1β production. Adipose tissue SVF from WT HF mice secreted significantly higher amounts of ATP-induced IL-1β compared with that from Fat-1 HF mice, whereas adipocytes produced negligible levels of IL-1β (Figure 3A). Because IL-1β production depends on caspase-1 activity, caspase-1 activity levels were then determined in adipose tissue SVF. As shown in Figure 3B, ATP treatment significantly activated caspase-1 in SVF from WT HF mice compared with that from Fat-1 HF mice. We further assessed whether IL-1β is also secreted from preadipocytes isolated from SVF. As shown in Figure 3C, preadipocytes from WT HF mice displayed enhanced IL-1β levels in response to ATP as observed in SVF, compared with those from Fat-1 HF mice. Additionally, a significant increase in caspase-1 activity was observed in preadipocytes from WT HF mice compared with those from Fat-1 HF mice (Figure 3D). We further assessed whether NLRP3 inflammasome is responsible for IL-1β secretion in preadipocytes as observed in adipose tissue. As shown in Figure 3E, MCC950, the NLRP3 inflammasome inhibitor, significantly suppressed ATP-induced IL-1β secretion in WT HF-derived preadipocytes to an extent comparable to that in Fat-1 HF-derived preadipocytes.

### 2.4. Major n-6 and n-3 Fatty Acid Composition and n-6/n-3 Ratio in Adipose Tissue and Preadipocytes

**Figure 4:**
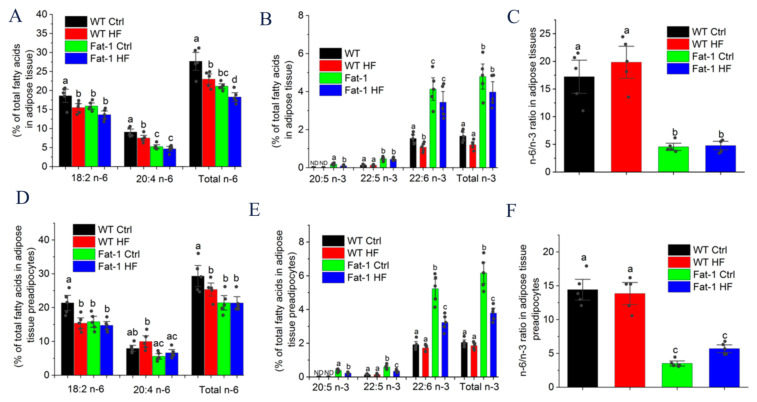
Major n-6 and n-3 PUFAs and the ratio of n-6/n-3 PUFAs in the adipose tissue or adipose tissue preadipocytes of WT and fat-1 mice fed a HF or Ctrl diet: (**A**) Major n-6 PUFAs in adipose tissue; (**B**) Major n-3 PUFAs in adipose tissue; (**C**) The ratio of n-6/n-3 PUFA in adipose tissue; (**D**) Major n-6 PUFAs in adipose tissue preadipocytes; (**E**) Major n-3 PUFAs in adipose tissue preadipocytes; (**F**) The ratio of n-6/n-3 PUFA in adipose tissue preadipocytes. Results are presented as mean ± SEM. Means not sharing a common superscript letter are significantly different at *p* < 0.05.

As shown in Figure 4A,B, the fatty acid composition of the total lipids extracted from adipose tissues showed distinct lipid profiles between fat-1 and WT mice. Significantly higher levels of n-3 PUFAs, including EPA (20:5 n-3), docosapentaenoic acid (DPA) (22:5 n-3), and DHA (22:6 n-3), whereas much lower concentrations of arachidonic acid (AA; 20:4 n-6) were observed in adipose tissues from fat-1 mice than from WT littermates regardless of whether the mice fed a Ctrl or HF diet. Of note, DHA was the most abundant endogenously synthesized n-3 PUFA in fat-1 mice. Additionally, the endogenous n-6/n-3 PUFA ratio in adipose tissue was significantly lower in Fat-1 Ctrl (4.56 ± 0.43) and Fat-1 HF mice (4.78 ± 0.51) than in WT Ctrl (17.22 ± 2.00) and WT HF mice (19.83 ± 1.93) whatever they fed a Ctrl or HF diet (Figure 4C). Similar data were also observed in adipose tissue preadipocytes (Figure 4D–F).

### 2.5. Endogenous n-3 PUFA Suppressed NLRP3 Inflammasome Signaling in Adipose Tissue

**Figure 5:**
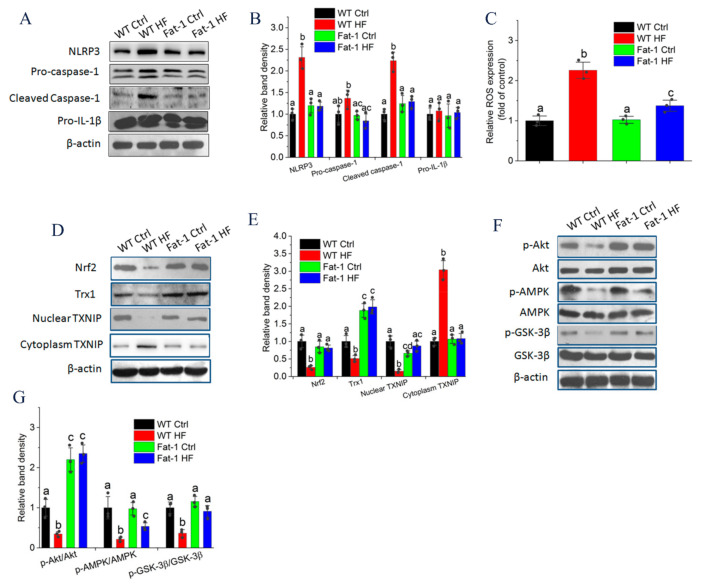
Endogenous n-3 PUFAs suppressed NLRP3 inflammasome activation by modulating AMPK/Akt/GSK-3β/TXNIP pathway: (**A**) The adipose tissue protein expression of NLRP3 inflammasome pathway such as NLRP3, pro-caspase-1, cleaved caspase-1 and pro-IL-1β was monitored by Western blotting analysis; (**B**) Band density analysis of western blotting bands in (**A**); (**C**) Adipose tissue ROS production was determined using the fluoroprobe DCFH-DA; (**D**) The protein expression of the Nrf2/Trx1/TXNIP pathway in adipose tissue was detected by western blotting analysis; (**E**) Band density analysis of western blotting bands in (**D**); (**F**) The protein expression of the AMPK/Akt/GSK-3β pathway was detected by western blotting analysis; (**G**) Band density analysis of western blotting bands in (F); Results are presented as mean ± SEM. Means not sharing a common superscript letter are significantly different at *p* < 0.05.

To bring insight into the benefits of endogenous n-3 PUFA on impairing IL-1β production via controlling NLRP3 inflammasome activation, we assessed the NLRP3 inflammasome pathway proteins by immunoblotting. As shown in Figure 5A,B, the HF diet significantly induced the protein expression of NLRP3, pro-caspase-1 and cleaved caspase-1 in adipose tissue of WT mice, whereas these proteins were comparable among that of WT Ctrl, Fat-1 Ctrl and Fat-1 HF mice. Interestingly, although the HF diet induced *IL-1β* mRNA expression compared with the Ctrl diet (Figure 2D), no difference in pro-IL-1β protein expression was observed whether the mice were fed a Ctrl or HF diet (Figure 5A). In addition, previous adipose tissue culture showed that endogenous n-3 PUFAs in fat-1 mice markedly inhibited HF diet-induced IL-1β secretion (Figure 2C). These results suggested that endogenous n-3 PUFAs impaired IL-1β production by inhibiting NLRP3 inflammasome activation.

### 2.6. Endogenous n-3 PUFAs Antagonized HF Diet-Induced IL-1β Secretion via AMPK/Akt/GSK-3β/TXNIP Axis

Because reactive oxygen species (ROS) are proximal signals for inflammasome activation [23], we determined the ROS levels in adipose tissue. ROS were significantly elevated in adipose tissue from WT HF mice (2.25 ± 0.11) compared with from Fat-1 HF mice (1.37 ± 0.07) (Figure 5C). Due to the key role of ROS in modulating NLRP3 inflammasome activation, we detected whether endogenous n-3 PUFAs suppressed ROS production and NLRP3 inflammasome activation via the Nrf2/Trx1 antioxidant axis. As shown in Figure 5D,E, Nrf2 and Trx1 protein levels were significantly down-regulated in adipose tissue from WT HF mice, whereas these two proteins were restored in adipose tissue from Fat-1 HF mice. Under oxidative stress conditions, increased ROS facilitate Trx1-TXNIP dissociation, then promoting TXNIP-NLRP3 interaction and inducing IL-1β production [24]. As shown in Figure 5D,E, the HF diet significantly up-regulated cytoplasmic TXNIP protein levels accompanied by decreased nuclear TXNIP levels in adipose tissue from WT HF mice, but not in adipose tissue from Fat-1 HF mice. Previous studies demonstrated that the Nrf2 activity is inhibited by GSK-3β, whereas the inhibitory activity of GSK-3β was blocked by being phosphorylated by Akt directly or AMPK indirectly [25,26]. As shown in Figure 5F,G, the HF diet markedly decreased the phosphorylation of Akt, AMPK and GSK-3β in adipose tissue of WT HF mice, whereas AMPK phosphorylation was partially and GSK-3β phosphorylation was completely restored in adipose tissue of Fat-1 HF mice. Meanwhile, Akt phosphorylation was significantly upregulated in the adipose tissue of fat-1 mice compared with that of WT mice.

### 2.7. Akt Phosphorylation Modulated by DHA and AMPK Controlled GSK-3β Activity and IL-1β Secretion

**Figure 6:**
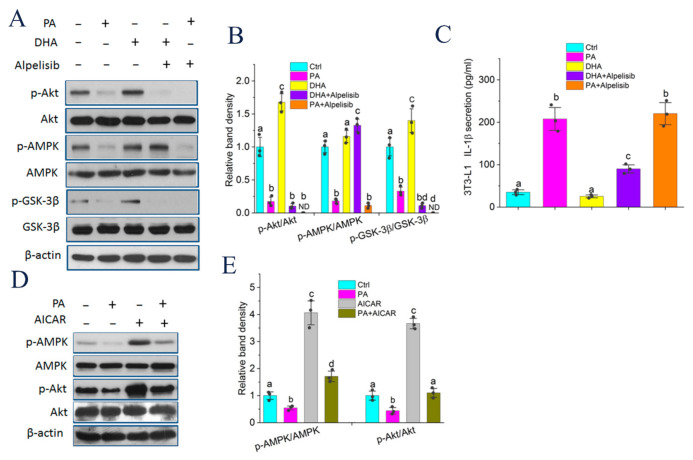
Akt phosphorylation modulated by DHA and AMPK controlled GSK-3β activity and IL-1β secretion: (**A**) 3T3-L1 preadipocytes were treated with LPS (10 ng/mL) for 3 h, and then treated with PA (250 μmol/L), DHA (20 μmol/L) or Alpelisib (5 nmol/L) for 24 h. Protein lysates were harvested for the analysis of p-Akt, Akt, p-AMPK, AMPK, p-GSK-3β, and GSK-3β protein expression; (**B**) Band density analysis of western blotting bands in (**A**); (**C**) The above-mentioned media in (**A**) was used for analysis of IL-1β secretion by ELISA; (**D**) 3T3-L1 preadipocytes were treated with LPS (10 ng/mL) for 3 h, and then treated with PA (250 μmol/L) or AICAR (2 mmol/L) for 24 h. Protein lysates were harvested for the analysis of p-Akt, Akt, p-AMPK, and AMPK protein expression; (**E**) Band density analysis of western blotting bands in (**D**). Means not sharing a common superscript letter are significantly different at *p* < 0.05. ND, not detectable.

Because IL-1β was secreted from nonadipocytes of adipose tissue such as preadipocytes (Figure 3C), we next explored the underlying mechanisms by which endogenous n-3 PUFAs antagonize HF diet-mediated NLRP3 inflammasome activation using 3T3-L1 murine preadipocytes. PA is the main saturated fatty acid in plasma and the HF diet, and DHA is the most abundant n-3 PUFA in adipose tissue of fat-1 mice. Therefore, these two fatty acids were used in the following mechanistic studies. To confirm whether reduced GSK-3β phosphorylation in vivo by HF diet can be restored by endogenous n-3 PUFA-mediated Akt phosphorylation in adipose tissue from Fat-1 HF mice, 3T3-L1 preadipocytes cultured in vitro were treated with PA or DHA alone or in combination with PI3K inhibitor alpelisib. As shown in Figure 6A,B, the phosphorylation levels of AMPK, Akt and GSK-3β were significantly reduced with PA treatment, whereas AMPK, Akt and GSK-3β were unchanged. In addition, DHA-treated 3T3-L1 cells exhibited higher Akt phosphorylation levels compared with Ctrl or PA-treated cells, consistent with those findings in adipose tissue. As expected, alpelisib treatment markedly abrogated DHA-mediated Akt phosphorylation. Correspondingly, GSK-3β phosphorylation was significantly suppressed in alpelisib-treated cells and remained comparable to that in PA-treated cells, suggesting GSK-3β phosphorylation is tightly regulated by Akt activity. However, AMPK phosphorylation was hardly affected by alpelisib treatment. Simultaneously, alpelisib treatment can rescue IL-1β secretion from 3T3-L1 preadipocytes in the presence of DHA (Figure 6C). We further assessed whether reduced Akt phosphorylation observed in adipose tissue of WT HF mice or in PA-treated cells was mediated by AMPK, 3T3-L1 preadipocytes were treated with PA or a widely used pharmacological AMPK activator, 5-aminoimidazole-4-carboxamide ribonucleoside (AICAR) alone or in combination. As shown in Figure 6D,E, AICAR reversed PA-mediated decreased phosphorylation levels of AMPK and Akt, suggesting that PA-mediated decreased AMPK activity, at least partly, contributes to reduced Akt phosphorylation.

### 2.8. GSK-3β Is Indispensable for Impairing PA-Induced NLRP3 Activity by DHA

**Figure 7:**
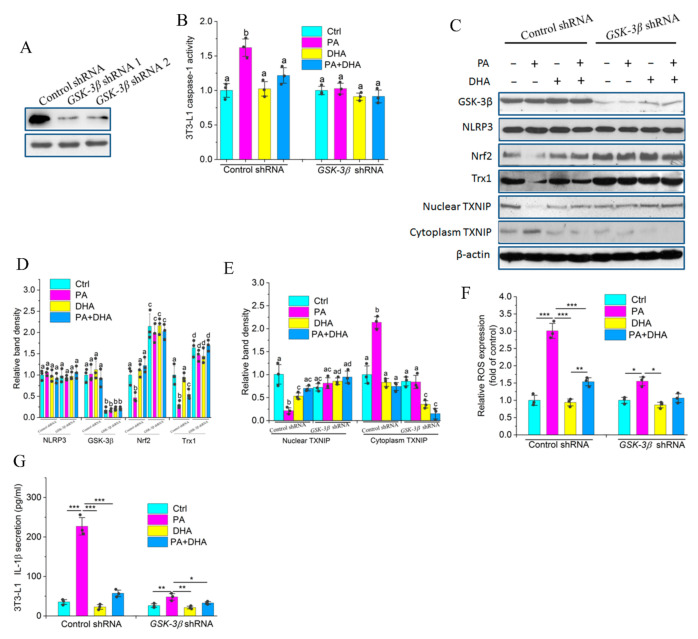
GSk-3β phosphorylation is indispensable for DHA-mediated inhibition of PA-induced IL-1β secretion: (**A**) 3T3-L1 preadipocytes were transfected with control shRNA or *GSK-3β* shRNA. The protein expression of GSK-3β was measured by western blotting; (**B**) 3T3-L1 preadipocytes transfected with control shRNA or *GSK-3β* shRNA were treated with LPS (10 ng/mL) for 3 h, and then stimulated with PA (250 μmol/L) or DHA (20 μmol/L) for 24 h. Caspase-1 activity was determined; (**C**) The cells with the above-mentioned treatment in (**B**) were harvested for the analysis for of NLRP3, Nrf2, Trx1, and TXNIP protein expression; (**D**,**E**) Band density analysis of western blotting bands in (**C**); (**F**) The cells with the above-mentioned treatment in (**B**) were harvested for analysis of ROS production using the fluoroprobe DCFH-DA; (**G**) The above-mentioned media in (**B**) was used for analysis of IL-1β secretion by ELISA. Results are presented as mean ± SEM. Means not sharing a common superscript letter are significantly different at * *p* < 0.05, ** *p* < 0.01 and *** *p* < 0.001.

The inhibitory effect of GSK3β on the Nrf2/Trx1 antioxidant pathway is reduced by its low expression or being phosphorylated by other kinases [27]. To assess whether GSK-3β plays a pivotal role in which DHA antagonizes PA-mediated NLRP3 inflammasome activity, we selectively knocked down GSK-3β expression using *GSK-3β* specific shRNAs. GSK-3β protein expression was significantly suppressed following transfection with the *GSK-3β* shRNAs, but not with the control shRNA (Figure 7A). PA-mediated caspase-1 activity was inhibited by DHA in 3T3-L1 preadipocytes transfected with control shRNA or by knockdown of GSK-3β in cells transfected with *GSK-3β* shRNA (Figure 7B). In addition, PA suppressed the protein expression of Nrf2 and Trx1, which was reversed by DHA or by knockdown of GSK-3β with *GSK-3β* shRNA, whereas the expression of NLRP3 was comparable across groups (Figure 7C,D). Simultaneously, PA-induced ROS and cytoplasmic translocation of TXNIP accompanied by increased IL-1β secretion were also abrogated by DHA or by knockdown of GSK-3β (Figure 7C,E–G). These results demonstrated that DHA suppresses PA-induced NLRP3 inflammasome activity by blocking the inhibitory activity of GSK-3β.

## 3. Discussion

Obesity is becoming an epidemic in both developed and developing countries. Obesity substantially increases the risk for a cluster of metabolic syndromes, particularly insulin resistance and T2D [28]. However, the underlying mechanism remains largely elusive. Previous studies suggested that obesity is associated with chronic low-grade inflammation in a variety of tissues, particularly adipose tissue, whereas chronic low-grade inflammation plays a causal role in the development of insulin resistance [29]. Therefore, chronic low-grade inflammation may serve as a causal link between obesity and insulin resistance. N-3 PUFAs, especially marine origin EPA and DHA, possess anti-inflammation properties and are attractive candidates for their therapeutic potential in many inflammation-driven human diseases [30]. In the present study, HF diet-induced glucose intolerance and insulin resistance were significantly attenuated by high endogenous n-3 PUFA in fat-1 mice despite HF diet-induced weight gain in fat-1 mice. Endogenous n-3 PUFAs exerted their beneficial effects via impairing HF diet-induced NLRP3 inflammasome activation in adipose tissue, especially in adipose tissue preadipocytes.

A number of studies suggest that IL-1β is implicated in the development of insulin resistance in diabetes mellitus [31,32] The notion is supported by the finding that blocking of IL-1 signaling pathways in insulin-resistant patients with diabetes mellitus by treatment with IL-1 receptor antagonist Anakinra resulted in sustained improvement in insulin sensitivity [33]. In agreement, our results demonstrated that HF diet induced-insulin resistance was accompanied by increased *IL-1β* mRNA expression and mature IL-1β secretion in adipose tissue of WT mice, which was reversed by enrichment of endogenous n-3 PUFAs in fat-1 mice (Figure 2C,D). In line with our results, Bellenger et al. reported that endogenous n-3 PUFAs inhibit *IL-1β* mRNA expression in pancreatic tissue from streptozotocin (STZ)-treated fat-1 mice which maintained lower glucose levels compared with STZ-treated wild type littermates [34]. Adipose tissue is a heterogeneous tissue composed of adipocytes and nonadipocytes such as preadipocytes and immune cells in its SVF. Numerous inflammatory cytokines such as tumor necrosis factor α are secreted from adipocytes and nonadipocytes [35,36]. In the present study, we found that IL-1β was mainly released from SVF and preadipocytes isolated from SVF in response to ATP, but not from adipocytes of adipose tissue (Figure 3A,C). This finding suggests that IL-1β secreted from preadipocytes of adipose tissue, is at least partially implicated in the development of HF diet-induced insulin resistance. Of note, immune cells from SVF could also be involved in controlling IL-1β release and promoting HF diet-mediated insulin resistance. Consistent with our results, Chung et al. reported that preadipocytes, but not adipocytes mediated LPS-induced inflammation and insulin resistance in primary cultures of newly differentiated human adipocytes [35]. However, most previous studies estimated that IL-1β secreted from macrophages mediates macrophage-adipocyte cross-talk and impairs insulin signaling in human primary adipocytes [37,38], which might overlook the effect of pro-inflammatory cytokines released from preadipocytes on insulin resistance.

In the present study, HF diet-induced IL-1β release from adipose tissue or adipose tissue preadipocytes was markedly suppressed by NLRP3 inflammasome inhibitor MCC950 or endogenous n-3 PUFAs, suggesting that endogenous n-3 PUFAs repress IL-1β secretion via inhibiting NLRP3 inflammasome activation. In line with our results, Yan et al. reported that exogenous DHA or EPA suppressed NLRP3 inflammasome-mediated IL-1β release in macrophages and prevented obesity-induced insulin resistance [39]. Interestingly, although the HF diet induced weight gain in fat-1 mice, it failed to induce IL-1β release, coincident with lower caspase-1 activity, reduced protein expression of NLRP3, pro-caspase-1, cleaved caspase-1 (Figure 1A, Figure 2C and Figure 5A). Consistent with our results, Finucane et al. found that monounsaturated fatty acids in the HF diet reduced IL-1β secretion and attenuated HF diet-induced insulin resistance despite obesity [40]. In addition, our results showed that the HF diet induced *pro-IL-1β* mRNA expression, but not pro-IL-1β protein expression. This could be largely explained by the key role of post-transcriptional regulation in modulating pro-IL-1β protein expression.

Redox signaling is tightly associated with various inflammatory conditions and innate immunity. ROS have been proposed to play an important role in triggering the NLRP3 inflammasome activation [23]. Here we demonstrated that the HF diet induced significantly elevated ROS in WT mice compared with fat-1 mice (Figure 5C), similar results were also observed in PA and/or DHA-treated 3T3-L1 preadipcytes (Figure 7F), suggesting that HF diet-induced NLRP3 inflammasome activation is tightly controlled by HF diet-induced ROS. HF diet-induced ROS might be owing to the inhibition of the Nrf2/Trx1 antioxidant pathway as evidenced by decreased Nrf2 and Trx1 protein levels, whereas endogenous PUFAs restored Nrf2 and Trx1 levels and suppressed ROS generation. TXNIP, which interacts with Trx1, one of the key regulators of the Nrf2/Trx1 redox signaling pathway under homeostatic conditions, is liberated by ROS, and in turn, binds to NLRP3 inflammasome resulting in its assembly and activation [24]. In agreement, our results showed that endogenous n-3 PUFAs inhibited HF diet-induced TXNIP cytoplasm translocation and subsequent NLRP3 inflammasome activation and IL-1β release (Figure 5D). In agreement with our results, Wang et al. reported that Nrf2 plays a role in DHA/EPA suppression of LPS-induced inflammation [41]. Previous studies reported that GSK-3β phosphorylates Nrf2 and blocks its role in preserving redox homeostasis in response to oxidant insults, whereas GSK-3β loses its inhibitory effect on Nrf2 via being phosphorylated by Akt [25]. Additionally, AMPK plays a vital role in controlling GSK-3β phosphorylation via regulating Akt activity or suppressing the dephosphorylation of GSK-3β [26]. In our study, the HF diet repressed phosphorylation of AMPK and Akt accompanied by reduced GSK-3β phosphorylation in WT mice. Importantly, endogenous n-3 PUFAs enhanced Akt phosphorylation and restored the GSK-3β phosphorylation in Fat-1 HF mice (Figure 5F). This could be explained that DHA promotes accumulation of membrane phosphatidylserine, which facilitates faster membrane translocation and phosphorylation of Akt [42]. We indeed found that DHA significantly increased Akt phosphorylation in 3T3-L1 cells, whereas PI3K inhibitor alpelisib markedly blocked Akt phosphorylation accompanied by decreased GSK-3β phosphorylation and attenuated the inhibitory effects of DHA on IL-1β production (Figure 6A). In this study, our results also suggest that AMPK regulates GSK-3β phosphorylation, at least partially through controlling Akt activation. Of note, the involvement of AMPK in suppressing the dephosphorylation of GSK-3β cannot be excluded. However, how AMPK modulates Akt phosphorylation as well as dephosphorylation of GSK-3β remains largely unknown and needs to be further investigated. In addition, the different effects of PA and DHA on IL-1β release were markedly abrogated in *GSK-3β* shRNA-transfected 3T3-L1 preadipocytes (Figure 7G), suggesting that GSK-3β functions as a pivot between AMPK, Akt, and the Nrf2/Trx1 antioxidant pathway, which tightly regulates inflammasome activation and IL-1β secretion.

## 4. Materials and Methods

### 4.1. Animals and Diet

All animal studies were performed in accordance with the use and care of laboratory animals and approved by the Ethics Committee of Ningbo University (Ningbo, China). The fat-1 transgenic mice were kindly provided by Dr. Jing X. Kang at Massachusetts General Hospital and Harvard Medical School (Boston, Massachusetts, the United States of America). Six-week-old male heterozygous fat-1 (*n* = 24) and non-transgenic littermate controls (WT, *n* = 24) were fed a control (Ctrl) diet or a HF diet for 4 months until sacrifice. Mice were maintained under specific pathogen-free conditions in standard cages in temperature- and humidity-controlled conditions with a 12 h light/dark cycle. The Ctrl diet contained (g/100 g diet) 4.5 g sucrose, 18.6 g casein, 8.6 g cellulose, 50 g wheat starch, 0.3 g DL-methionine, 7 g mineral mix, 1 g vitamin mix, and 10 g safflower oil. The HF diet contained (g/100 g diet) 4.5 g sucrose, 18.6 g casein, 8.6 g cellulose, 15 g wheat starch, 35 g lard, 0.3 g DL-methionine, 7 g mineral mix, 1 g vitamin mix, and 10 g safflower oil.

### 4.2. Glucose and Insulin Tolerance Test

Oral glucose tolerance test (OGTT) and insulin tolerance test (ITT) were performed as described previously [40]. Briefly, for the OGTT, mice were fasted for 16 h, followed by a gavage of glucose (1.5 g kg^−1^). For the ITT, mice were fasted for 4 h and injected intraperitoneally with insulin at a final concentration of 0.75 U/kg body weight. Blood glucose concentrations were determined immediately at the indicated time points (i.e., at 0, 30, 60, 90 and 120 min) before and after a glucose/insulin challenge with a commercial glucose meter.

### 4.3. Tissue Culture

Epididymal adipose tissues were isolated and washed in cold PBS. These tissue fragments were then cultured in 12-well plates in opti-MEM medium (100 mg/mL) supplemented with 1% penicillin/streptomycin. After 24 h, culture media was collected and analyzed for IL-1β and IL-18 according to the manufacturer’s instructions.

### 4.4. Stromal Vascular Fraction, Mature Adipocyte and Preadipocyte Fractionation and Culture

Primary stromal vascular fraction (SVF) and adipocytes were fractionated from white adipose tissue from 22-week-old fat-1 or WT mice as described previously [43]. Epididymal adipose tissues from fat-1 or WT mice were isolated, minced, and digested by type II collagenase (2 mg/mL) in Krebs–Ringer bicarbonate buffer containing 10 mM HEPES (pH7.4) and 5% FBS for 30 min at 37 °C with shaking. The collagenase digests were filtered through a 200-mesh strainer and centrifuged at 300× *g* for 5 min to separate suspended adipocytes and SVF. The SVF cells (1 × 10^6^ cells/mL), and adipocytes (200 μL packed volume/mL) were seeded, cultured in DMEM containing 10% FBS, and 1% penicillin/streptomycin and treated with or without ATP for 24 h. Protein lysates and culture media were prepared for further analysis. Separately, in order to separate preadipocytes from adipose tissue, adipose tissue was digested by type II collagenase (2 mg/mL) in Krebs–Ringer bicarbonate buffer containing 10 mM HEPES (pH7.4) and 5% FBS at 37 °C for least 1 h with shaking. The collagenase digests were centrifuged at 350× *g* for 5 min to obtain sedimented stromal cells. These stromal cells were then suspended in an erythrocyte-lysing buffer for 5 min, washed and seeded in DMEM containing 10% FBS. Nonadherent cells were removed after 16–20 h for cell attachment.

### 4.5. Cell Culture

3T3-L1 pre-adipocytes were purchased from the American Type Culture Collection (Manassas, VA, the United States of America) and cultured in Dulbecco’s modified Eagle’s Medium (DMEM) supplemented with 10% foetal bovine serum (FBS), 1% penicillin/streptomycin.

### 4.6. Measurement of ROS of Adipose Tissue and Cultured 3T3-L1 Preadipocytes

Adipose tissue from WT or fat-1 mice was isolated, minced, and digested by type II collagenase (2 mg/mL) in PBS for 30 min at 37 °C with shaking. A total of 5% FBS was then added into the collagenase digests to terminate the digestion process. The collagenase digests were filtered through a 200-mesh strainer to remove tissue blocks. The cell suspension or cultured 3T3-L1 preadipocytes were then incubated with 50 μM of DCFH-DA (Beyotime, Jiangsu, China) at 37 °C in a dark place for 30 min. DCF fluorescence intensities were detected by a fluorescence microplate reader (Thermo Fisher Scientific, Massachusetts, MA, USA).

### 4.7. Real-Time PCR Analysis

For real-time PCR analysis, total RNA was extracted from adipose tissue using TRIzol reagent (Invitrogen, Carlsbad, CA, USA) in accordance with the manufacturer’s instructions. A total of 1 μg RNA was transcribed to complementary DNA using a reverse transcriptase system (Bio-Rad, Hercules, CA, USA). RT-PCR was performed on an Agilent Mx3000P QPCR System (Agilent, CA, USA) with SYBR Green Supermix (Bio-Rad, Hercules, CA, USA). The following primer was used in this study: *IL-1β* (forward 5′-CTCACAAGCAGAGCACAAGC-3′, reverse 5′-CTCAGTGCAGGCTATGACCA-3′).

### 4.8. Transfection of GSK-3β shRNA

Control shRNA and *GSK-3β* shRNA lentiviruses were obtained from Xizhao Biotech (Shanghai, China). 3T3-L1 preadipocytes were transfected with control shRNA and *GSK-3β* shRNA lentiviruses using 5 μg/mL polybrene transfection reagent according to the protocol of the shRNA transfection kit (Xizhao Biotech, Shanghai, China). After 72 h, cells were harvested, and western blotting analysis was used to determine GSK-3β expression.

### 4.9. Western Blotting Analysis

Adipose tissues isolated from mice were homogenized in ice-cold RIPA buffer without Triton X-100. After low-speed centrifugation (6000× *g* at 4 °C) for 15 min, the fat cake was removed, and Triton X-100 was added to a final concentration of 1% (*v*/*v*). 3T3-L1 preadipocytes were directly homogenized and lysed in Triton X-100 containing buffer. The extracts were sonicated and centrifuged at 12,000× *g* at 4 °C for 15 min. Protein concentrations were determined by a bicinchoninic acid (BCA) protein assay kit (Biyuntian Biotech, Haimen, China). Protein extracts were separated by 10% SDS-PAGE and transferred onto nitrocellulose membranes (Millipore, Massachusetts, MA, USA). Membranes were blocked in 5% fat-free milk at room temperature for 1 h and incubated with primary antibodies overnight at 4 °C, followed by incubation with the HRP-conjugated secondary antibodies for 1 h at room temperature. Proteins were visualized by chemiluminescence reagent ECL. The protein band densities were quantified using Image-Pro Plus 6.0 (IPP 6.0) software (Media Cybernetics, Massachusetts, MA, USA). The primary antibodies used in this study: rabbit anti-pro-IL-1β, rabbit anti-IL-1β, rabbit anti-pro-caspase-1, rabbit anti-cleaved caspase-1, rabbit anti-TXNIP, rabbit anti-Akt, rabbit anti-phosphor-Akt (Thr308), rabbit anti-NLRP3, rabbit anti-AMPK, rabbit anti-phosphor-AMPK (Thr172), rabbit anti-GSK3β, rabbit anti-phosphor-GSK3β (Ser9), rabbit anti-Nrf2, rabbit anti-Trx1, mouse anti-β-actin, and the HRP-conjugated secondary antibodies were purchased from Cell Signaling Technology (Danvers, MA, USA).

### 4.10. Caspase-1 Activity Assay

The caspase-1 activity in the lysates of SVF, 3T3-L1 preadipocytes and adipose tissue preadipocytes was determined using a caspase-1 fluorometric kit (Biovision, San Francisco, CA, USA) as previously described [44]. Cells were lysed in hypotonic cell lysis buffer on ice for 10 min and centrifuged to remove the insoluble fraction (12,000× *g*, 10 min). A total of 50 μL SVF lysate was incubated with 50 μM peptide YVAD-AFC and reaction buffer (50 μL) for 2 h. The fluorescence of the cleaved substrate was measured using a fluorometer at set time intervals.

### 4.11. The Fatty Acid Composition of Adipose Tissue and Adipose Tissue Preadipocytes

The fatty acid composition in adipose tissues and adipose tissue preadipocytes was determined by gas chromatography, as described previously [34].

### 4.12. Histological Analysis

For histological investigations, adipose tissues were fixed in 4% paraformaldehyde in phosphate buffer overnight and then embedded in paraffin for hematoxylin and eosin (H&E) staining.

### 4.13. Statistical Analysis

Data were expressed as mean ± SEM and statistical differences were evaluated by one-way ANOVA followed by Newman–Keuls test. Statistical significance was presented as *p* < 0.05.

## 5. Conclusions

**Figure 8:**
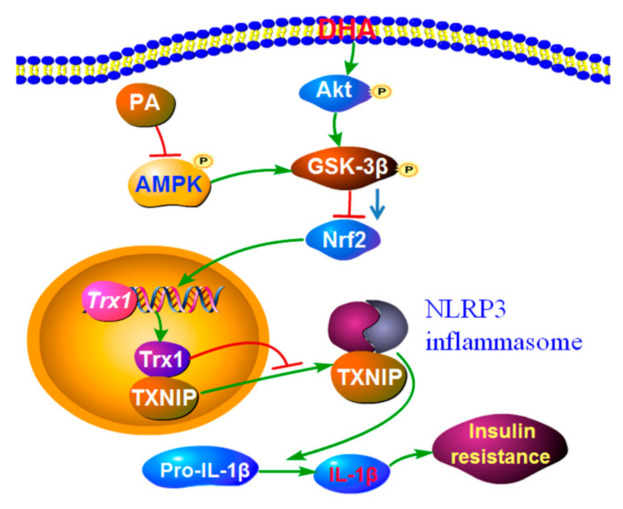
Schematic depicting the underlying mechanism by which endogenous n-3 PUFAs, especially DHA antagonize HF diet-induced insulin resistance. Mechanistically, PA, the main saturated fatty acid in HF diet inactivated AMPK and led to decreased GSK-3β phosphorylation, at least partially through reducing Akt activity, which ultimately blocked the Nrf2/Trx1 antioxidant pathway and induced TXNIP cytoplasm translocation and NLRP3 inflammasome activation, whereas DHA, the most abundant n-3 PUFA in fat-1 adipose tissue, reversed this process via inducing Akt activation. Blue arrow indicates the inhibitory activity of GSK-3β is weakened when GSK-3β (Ser9) is phosphorylated.

As summarized in Figure 8, our results demonstrated that HF diet-induced insulin resistance was ameliorated by endogenous n-3 PUFAs via inhibition of HF diet-induced NLRP3 inflammasome activation and IL-1β release in adipose tissue, especially in adipose tissue preadipocytes. Mechanistically, PA, the main saturated fatty acid in the HF diet inactivated AMPK and led to decreased GSK-3β phosphorylation, at least partially through reducing Akt activity, which ultimately blocked the Nrf2/Trx1 antioxidant pathway and induced TXNIP cytoplasm translocation and NLRP3 inflammasome activation, whereas DHA, the most abundant n-3 PUFA in fat-1 adipose tissue, reversed this process via inducing Akt phosphorylation. Our study emphasizes that the inhibitory activity of n-3 PUFAs on inflammasome activation is important for their beneficial effects on attenuating obesity-associated insulin resistance. In addition, given the involvement of inflammasome in the development of most inflammatory diseases, n-3 PUFAs might have potential clinical use in the prevention or adjuvant treatment of inflammation-driven diseases [45].

## Data Availability

Raw data available upon reasonable request.

## Sample Availability

Samples of the compounds are available from the authors.
